# Editorial for the Special Issue on Nanomaterials for Micro/Nanodevices

**DOI:** 10.3390/mi15101245

**Published:** 2024-10-10

**Authors:** Amir Hussain Idrisi

**Affiliations:** Mechanical and Aerospace Engineering, Michigan Technological University, Houghton, MI 49931, USA; amirhus@mtu.edu

The Special Issue of *Micromachines*, titled “Nanomaterials for Micro/Nanodevices”, comprehensively examines the intersection of nanotechnology and micro/nanodevices. This issue is a collection of ten papers that explores the cutting-edge developments and applications of nanomaterials in the fabrication and performance enhancement of micro- and nano-scale devices. As the demand for smaller, faster, and more efficient electronic and mechanical systems grows, nanomaterials play a pivotal role in revolutionizing device engineering [1]. Nanomaterials offer unique electrical [2], mechanical [3], and chemical [4] properties that make them ideal for pushing the boundaries of micro/nanodevice performance. By advancing the interface between materials science and nanotechnology, this issue showcases innovative research driving the future of micro-/nano-scale technologies. Additionally, it addresses challenges such as scalability, cost-effective manufacturing, and material–device compatibility. These featured studies provide insights into current limitations that nanomaterials can address through interdisciplinary collaboration, setting the stage for next-generation innovations in electronics, energy harvesting, and biomedical technologies.

The following contributions in this Special Issue cover a broad range of applications and technologies and address specific gaps in current knowledge:

The review article by Zhang et al. [5] explores the research on organic–inorganic hybrid thin-film packaging for flexible organic electroluminescent devices using plasma-enhanced atomic layer deposition (PEALD) and molecular layer deposition (MLD) techniques. The authors examine the methods and optimization strategies for preparing hybrid encapsulation layers, the importance of these devices in flexible electronics, and applications. The paper also evaluates the encapsulation effect, stability, and reliability of these layers, summarizing current progress and outlining future research directions and trends.

The exploration of bio-inspired nanomaterials (BINMs) is a significant advancement in the biomedical applications of micro/nanodevices discussed by Harun-Ur-Rashid et al. [6]. These nanomaterials offer exceptional biocompatibility and multifunctionality, with potential in biosensors, drug delivery, and tissue engineering. This review examines various BINMs from proteins, DNA, and biomimetic polymers, their integration into devices, and their impacts on the biomedical field. It also addresses challenges and proposes strategies to enhance efficiency and reliability, aiming towards maximizing the applications of BINMs in advanced biomedical devices.

Complementing the focus on BINMs, the issue also delves into surface engineering techniques that can further enhance device functionality. One such technique is the development of superhydrophobic surfaces, which have garnered significant attention for their versatile applications. A review article on superhydrophobic surfaces discussed by Barthwal et al. [7] comprises theoretical foundations, fabrication methods, applications, and challenges. Key principles such as Young’s equation, Wenzel and Cassie–Baxter states, and wetting dynamics are examined. This article also highlights various fabrication techniques and applications in fields like healthcare and environmental protection. Furthermore, it addresses challenges such as durability, scalability, and environmental concerns, aiming to guide future research and innovation in this field.

Transitioning from theoretical reviews to experimental investigations, the Special Issue includes cutting-edge research on novel materials. A valuable insight into the synthesis, characterization, and possibility for the use of nanostructured spinel chromites in advanced capacitor technologies was discussed by Mykhailovych et al. [8]. This study reports the successful synthesis of dielectric ZnCr_2_O_4_ nanoparticles using a sol–gel auto-combustion method followed by heat treatments in air from 5 to 11 h, in air at 500 to 900 °C. The morphology of the nanoparticles was analyzed using various techniques, revealing that higher temperatures increased nanoparticle size from 10 to 350 nm and changed their shape to pseudo-octahedra. These larger nanoparticles exhibited high dielectric constants and low dielectric losses. The annealing temperatures, which resulted in the formation of single-phase ZnCr_2_O_4_ nanopowders, range between 700 °C and 900 °C and the annealing time is 7 h, whereas lower annealing temperatures resulted in the formation of a secondary phase, identified as Cr_2_O_3_. However, a well-defined octahedral morphology of the nanoparticles is obtained at an annealing temperature of 900 °C for at least 11 h.

Expanding on the theme of material enhancement, Halford et al. [9] investigated the use of (3-Aminopropyl) triethoxysilane (APTES) as a primer for aluminum alloy AA2024-T3 and focused on its stability and interaction with a polystyrene (PS) topcoat. The aluminum alloy samples were primed with APTES under various durations of concentrated vapor deposition (20, 40, or 60 min). The samples were optional post-heat treatment and/or PS topcoat and were comparatively characterized with the help of electrochemical impedance spectroscopy (EIS) and surface energy. Optimal corrosion resistance was achieved with a 40 min APTES primer and post heat treatment, attributed to increased surface energy and enhanced PS wettability. The results also suggest that a thinner APTES primer (deposited for 20 min) enhances protection against corrosion in the early stages of exposure to the corrosion solution. The findings highlight APTES’s potential in microdevice applications.

In research by Stine et al. [10], the authors aimed to adapt existing tools to identify nanoplastics and differentiate by polymer type using deformation data from scanning electron microscopy (SEM). Polyvinyl chloride (PVC), polyethylene terephthalate (PET), and high-density polyethylene (HDPE) were analyzed and compared against common environmental materials like algae and clay. Distinct deformation patterns of plastic particles were observed in selected environmental media and successfully developed a computer vision algorithm to enhance identification efficiency.

Turcitu et al. [11] compared the compliance and deformation properties of three different characteristic-sized (Chip A, Chip B, and Chip C, with parallel channel widths of 100, 40, and 20 μm, respectively) microfluidic devices made of polydimethylsiloxane (PDMS) and Norland Optical Adhesive (NOA). Further comparison was made based on properties like Young’s modulus, roughness, contact angle, deformation, flow resistance, and compliance. The results indicate that chip A was found to be 4 times longer for PDMS devices than NOA devices, 1.6 times for chip B, and 2.5 times for chip C. The modulus of elasticity was significantly higher for NOA (1743 MPa) compared to PDMS’s (2 MPa). The lower compliance and deformation of NOA indicate it as a better material for microfluidic device fabrication.

Almodóvar et al. [12] introduced a cost-effective method to produce high-performance cathodes for aluminum-air batteries. Commercial fuel cell cathodes were modified using the electrodeposition of nickel and manganese species. Optimal electrodeposition conditions were identified using Raman, SEM, TEM, and electrochemical characterization techniques, confirming the successful incorporation of these species. The modified cathodes showed enhanced electrochemical activity and achieved capacities of 50 mA h cm^−2^ for the electrodeposited Ni:Mn (3:2) cathode in aluminum-air batteries. This method is cost-effective and scalable to enhance the electrochemical performance. The results highlight its potential for practical applications in emerging energy storage technologies.

Yu et al. [13] synthesized CH_3_NH_3_PbI_3_/graphene (MG) materials using organic–inorganic hybrid perovskite (OIHP) and graphene. The phase composition, lattice morphology, and micro-morphology of MG materials (MG-1 to MG-5) with different proportions (24:1, 16:1, 12:1, 8:1, 6:1) were analyzed by XRD, Raman, PL, and SEM. It was found that for MG composites, MG materials at MG-1 and MG-2 ratios exhibited better electromagnetic wave (EMW) absorption performance than other ratios. The optimal component ratio of 16:1 resulted in an absorber thickness of 1.87 mm and an effective EMW absorption width of 6.04 GHz covering the Ku frequency band. The CH_3_NH_3_PbI_3_ component enhanced polarization loss, while graphene improved electrical conductivity loss. This research broadens the EMW absorption frequency band of OIHP and advances new EMW-absorbing materials.

In the last contribution, Wang et al. [14] employed sodium anthraquinone-2-sulfonate (AQS) with high redox reactivity to enhance the accessibility of ions and electrolyte and the capacitance performance of niobium carbide (Nb2C) MXene. The Nb2C–AQS composite shows significantly higher electrochemical capacitance (36.3 mF cm^2^) compared to pure Nb2C (16.8 mF cm^2^) at a scan rate of 20 mV s^−1^. The supercapacitors showed excellent flexibility and stability, with 99.5% capacitance retention after 600 cycles.

Overall, this Special Issue of *Micromachines* presents a thorough and insightful exploration of the current state of research in nanomaterials for micro/nanodevices. The diverse topics and innovative approaches covered in these papers underscore the vast potential of nanomaterials to drive future technological advancements across various fields. Researchers and practitioners can draw valuable insights from these studies to further enhance the development and application of nanomaterials in both technology and medicine.
